# “Drilling Down”: psychiatry and dentistry in collaborative action

**DOI:** 10.1192/bjo.2022.530

**Published:** 2022-06-20

**Authors:** Chiara Cattra, Elizabeth Gonzalez Malaga

**Affiliations:** 1 National Medical Director's Clinical Fellow, General Medical Council, London, United Kingdom; 2 Solent NHS Trust, Portsmouth, United Kingdom; 3 University of Southampton, Southampton, United Kingdom; 4 General Dental Council, London, United Kingdom

## Abstract

**Aims:**

People with severe mental health illnesses experience multiple adverse physical health outcomes, in part caused by difficulties accessing, engaging with health promoting behaviour, treatment and recovery strategies. As oral health is a major contributor to physical and mental wellbeing, obstacles to care, prevention, and therapy play an important role in the oral health of individuals with mental illnesses. Psychiatric medications are known to predispose to oral health pathologies, including sialorrhea and dental caries, electroconvulsive therapy (ECT) may rarely result in dental fractures, and substance misuse may contribute to poor dental health. Unsurprisingly, COVID-19 has been more acutely noticed by those already at risk of worse oral health outcomes, including people with mental health conditions.

**Methods:**

We address the interplay between mental health and dental care, outline evidence behind the vital importance of collaborative working, and advocate for a joint approach between mental health and dental teams utilising harm reviews adapted to assessing the impact of delay dental care upon patients and families’ quality of life. As COVID-19 persists and winter pressures are experienced every year, these raise the question of what needs to be done to demonstrate the effects of poor oral health on patients with learning difficulties and mental illness.

**Results:**

With annual winter pressures in healthcare, many elective operations are postponed to allow capacity for increased demand. Dental general anaesthetics are amongst the first lists to be suspended, particularly since the arrival of COVID-19. During the first peak of the pandemic, limited access to personal protective equipment and concerns over viral transmission risked by aerosol generating procedures restricted the provision of community dental care to urgent cases, and dental general anaesthetics to life-threatening infections alone.These impacts were particularly acute for those with learning difficulties and mental illness, further exacerbated by social, geographical and financial inequalities. Waiting for patients to deteriorate to access dental care treatment seems in direct opposition to the mental health movement towards community and early management of mental illnesses.

**Conclusion:**

Adapted harm reviews are a powerful tool for mental health and dental teams to demonstrate to hospital managers the multidimensional impact that poor oral health has and causing physical, behavioural and emotional deterioration on patients, families and supporting staff. Wider understanding of the dental needs of those with mental health conditions may foster research on the interplay between oral and psychological health, and remains vital to multidisciplinary, compassionate and holistic care.

